# Fast prototyping of a local fuzzy search system for decision support and retraining of hospital staff during pandemic

**DOI:** 10.1007/s13755-021-00150-y

**Published:** 2021-05-11

**Authors:** Evgeny A. Bakin, Oksana V. Stanevich, Daria M. Danilenko, Dmitry A. Lioznov, Alexander N. Kulikov

**Affiliations:** 1 Pavlov First Saint Petersburg State Medical University, L’va Tolstogo str. 6-8, Saint Petersburg, 197022 Russia; 2 Smorodintsev Research Institute of Influenza, Prof. Popov str. 15/17, Saint Petersburg, 197376 Russia

**Keywords:** Fuzzy search, COVID-19, Decision support algorithm, Patient-like-mine, Prototyping

## Abstract

**Purpose:**

The COVID-19 pandemic showed an urgent need for decision support systems to help doctors at a time of stress and uncertainty. However, significant differences in hospital conditions, as well as skepticism of doctors about machine learning algorithms, limit their introduction into clinical practice. Our goal was to test and apply the principle of ”patient-like-mine” decision support in rapidly changing conditions of a pandemic.

**Methods:**

In the developed system we implemented a fuzzy search that allows a doctor to compare their medical case with similar cases recorded in their medical center since the beginning of the pandemic. Various distance metrics were tried for obtaining clinically relevant search results. With the use of R programming language, we designed the first version of the system in approximately a week. A set of features for the comparison of the cases was selected with the use of random forest algorithm implemented in Caret. Shiny package was chosen for the design of GUI.

**Results:**

The deployed tool allowed doctors to quickly estimate the current conditions of their patients by means of studying the most similar previous cases stored in the local health information system. The extensive testing of the system during the first wave of COVID-19 showed that this approach helps not only to draw a conclusion about the optimal treatment tactics and to train medical staff in real-time but also to optimize patients’ individual testing plans.

**Conclusions:**

This project points to the possibility of rapid prototyping and effective usage of ”patient-like-mine” search systems at the time of a pandemic caused by a poorly known pathogen.

## Introduction

Recent events revealed that despite considerable progress in health information systems (HIS), biostatistics, clinical pharmacology and evidence-based medicine in general, humanity still does not have sufficient tools to address sudden pandemic outbreaks. The spread of the disease caused by a new coronavirus infection (COVID-19) was accompanied by frequent changes in clinical guidelines, absence of unified standards for anti-epidemic measures, and a huge number of publications, sometimes conflicting with one another, appearing every day. Besides, a few cases of medical data falsification were detected. All this increased uncertainty, as well as a high workload of medical personnel in COVID-19 centers [1].

To assist physicians, research groups in IT started developing decision support systems (DSS) to mitigate different aspects of the pandemic [2]. Thus, paper [3] investigates the issues of constructing a simple patient condition severity classifier based on remote survey data to adjust medical logistics. System [4] developed by Sapio Analytics is planned as a tool to optimize quarantine regime. Project Vida, proposed by a team of scientists from the MIT, aims to conduct a comprehensive analysis of the situation in a region (involving geographic information systems data) for the subsequent construction of SARS-CoV2 propagation models and assessment of its impacts on socio-economic indicators [5, 6]. Study [7] investigates the dependence of pandemic spread velocity on the measures of epidemic control. Similarly, in [8], mortality predictions are made in real time basing on current epidemic data. Various platforms for the remote provision of medical services via the Internet are also offered [9].

At the same time, the accumulation of inpatients data while in hospital allowed for a variety of forecasting models for predicting the likelihood of infection and the severity of disease basing on case history, demographic and clinical parameters as well as laboratory tests [10–15].

These models were conceived as a way to help physicians (including non-infectious specializations), operating in centers for the treatment of COVID-19. However, in spite of all the above mentioned factors, the effectiveness of these tools in clinical practice remains very limited [16]. We consider the following main reasons for delaying the implementation of the decision-making systems that are so necessary during a pandemic.

1. *Differences in hospital structure and equipment*
  Patient management practices, the severity of cases admitted to a hospital, the possibility for conducting expensive laboratory tests on a regular basis, congestion in intensive care units (ICUs) have a strong impact on the main endpoints of the above studies. Thus, forecasting models built in the context of large, well-equipped hospitals may not be applicable in typical conditions in regional clinics or clinics in developing countries, and vice versa.
2. *Significant percentage of non-infectionists working in COVID-19 treatment centers*
  Refurbishment of non-infection hospitals was often carried out to cope with the outbreak of cases. Specialists in other medical fields who had no experience in the treatment of infectious diseases were rapidly involved in the work. In addition to the direct supervision of patients, they simultaneously faced the need to bridge a knowledge gap. The simple use of computer predictors that form a binary response (e.g. ”death”/”recovery”) doesn’t contribute to the development of clinical skills, but, on the contrary, could introduce an element of formality in the doctor’s work.
3. *Lack of medications with a proven effectiveness*
  History has shown that during a rapid spread of a previously unknown infection, the standard practice of conducting clinical trials of medications has intolerably long lags. The off-label use of medications basing on results obtained *in vitro*, or in the absence of randomization and blinding often turned out to be ineffective, and, in some cases, even dangerous. An example is the widespread use of combinations of drugs based on hydroxychloroquine and ART at the initial stages of the pandemic, the expediency of which was then refuted [17, 18]. Therefore, informing the physician about a patient’s high risk of ICU admission (or death) in the absence of effective prevention therapy may cause unnecessary stress or, conversely, personnel passivity during treatment.
4. *Skeptical attitude to the published information*
  Unfortunately, the COVID-19 pandemic is also accompanied by a series of scandals related to unfair testing and falsification of results. The most noticeable of these cases concerns the activities of the Surgisphere Company, which was found to have provided unreliable medical data for several studies [19]. As a result, a few articles published in well-established journals The Lancet and The New England Journal of Medicine, were subsequently withdrawn [20, 21]. All this reduces the level of trust of medical professionals in the published data and makes them unwilling to rely on the experience of other organizations.
5. *Low usability of proposed approaches*
  As was shown before, a convenient graphical user interface is crucial for making the application attractive for every-day usage in a stressful environment [22]. Despite a few such apps developed recently (e.g. those presented in [23–25]), the lack of usability of COVID-19 decision support tools is still noticeable.

Thus, the attitude of clinicians to the published decision support systems can be reduced to the formula ”Abstract models based on irrelevant or falsified data urging to unknown actions”. This inspired us to implement a different medical informatics approach for helping physicians.

## Objectives

When developing a decision support system at Saint-Petersburg Pavlov University, we set the goal of creating an application with a graphical interface with the help of which a doctor, having entered a relatively small number of indicators of their patient, could find in the database (DB) the most similar cases recorded in the hospital earlier. Having studied the therapies applied earlier and what outcomes they have led to, the doctor can adjust the course of treatment and, if necessary, consult their colleagues who supervised previous patients. It is obvious that due to the uniqueness of each individual patient, the search based on the exact match of all clinically significant symptoms is hardly possible.

Hence, we decided to implement a fuzzy search system that allows incomplete data matching. Fuzzy search systems for medical data have been actively discussed recently in relation to computerization of medical institutions and the growth of stored information volume. Some of them are mainly focused on searching for patients based on incomplete or mistakenly entered identification data (full name, date of birth, sex, policy number, etc.) [26–29]. The others utilize the more sophisiticated approach named ”patient-like-mine”, which implies aggregating previous electronic health records for searching clinically-similar patients in a hospital history [30–32]. These systems may have quite a complex structure and require a deep integration with a local health information system (HIS). All this made it difficult to develop and introduce them into clinical practice immediately after the pandemic outbreak.

To address the challenge, we set the following tasks:

- Select software tools which allow fast prototyping and deployment in the local network by work of a biostatistician unit without the involvement of third parties and without a modification in the local HIS.
- Create and pre-process a dataset containing data for all the patients in the COVID-19 treatment center, including case data, demography, anthropometry and daily results of laboratory tests.
- Select a relatively brief list of patient features that contain a sufficient amount of information about a patient’s current condition.
- Develop a fuzzy search system that takes in the instant values of the current patient features entered by the doctor, and outputs a set of the most similar cases, indicating the ID numbers of past patients and the days of hospitalization when this similarity was noticed.
- Implement a graphical user interface (GUI) that represents the information found in a user-friendly form and allows the doctor to quickly navigate the cases under consideration.

## Methods

### Software tools

*R* version 3.6.3 was chosen as the main programming language [33]. The list of the main R packages used in the project is given in Table 1.

**Table 1:** **R** packages used in the project

Purpose	Packages	Reference
Data pre-processing	*tidyr*	[34]
Application of machine learning methods for main features selection	*caret*	[35]
Vizualization of health information data	*ggplot2* *ggpubr*	[36, 37]
Building of GUI	*shiny*	[38]

### Generation of initial dataset

From the local health information system (HIS), we downloaded 6 separate datasets which stored data about the patients’ states:

1. Registration data: the date of intake, the number of bed-days in the hospital, the treatment outcome.
2. Demographic and anthropometric data: age, sex, height, weight (combined later into BMI).
3. Anamnestic data: the number of days of illness, the presence of chronic diseases, the smoking status.
4. Results of biochemical tests: ALT, AST, amylase, bilirubin, bound bilirubin, creatinine, SRP, D-dimer, fibrinogen, ferritin, glucose, glucose ABB, lactate ABB, potassium, potassium ABB, sodium, sodium ABB, total protein, troponin I, urea.
5. Results of a blood differential test: total leukocytes, neutrophils, monocytes, platelets, lymphocytes, hemoglobin.
6. NEWS scale components: heart rate (HR), respiratory rate (RR), systolic blood pressure, temperature, saturation, need for additional oxygenation, AVPU score.

**Fig. 1:**
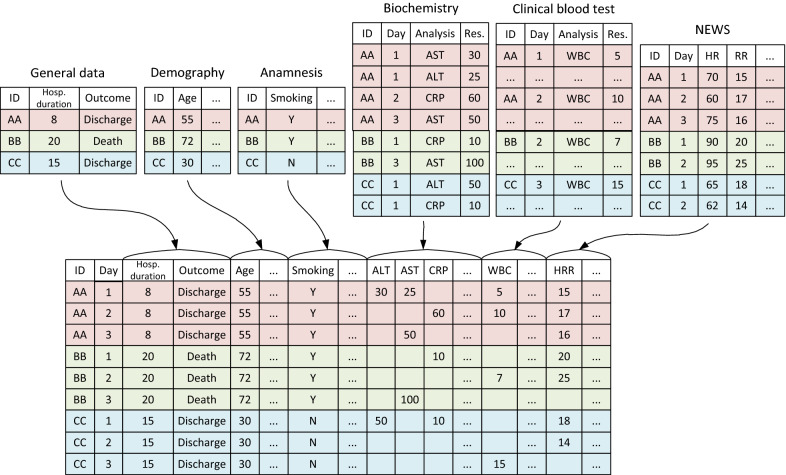
The principle of formation of the initial dataset

All the datasets contained the key field Patient ID. Datasets 4-6 were downloaded in a long format with reference to the date of the tests. Further, all the datasets were combined into a single array, one line of which corresponded to one day of hospitalization of one patient. The values in the columns corresponded to the results of tests taken on a particular day. If on one of the days a certain test was taken several times, the median of the measured values was entered in the table. The illustration of the process of forming the source dataset is shown in Figure 1. Next, missing values were filled in using last observation carried forward procedure (LOCF) [39].

### Features selection

For the selection of the main features which reflect the condition of the patient, we conducted training of a set of standard machine learning algorithms: logistic regression, random forest, k-nearest neighbors, support vector machine and gradient boosting. The results of the algorithms were checked using k-fold cross-validation. For the best classifier (in terms of area under the curve, AUC), the features were arranged according to their importance. Top 12 clinical and laboratory features were selected from this list, where sex and age were also added. Thus, the selection of similar cases was carried out basing on 14 features.

### Fuzzy search

To carry out the search, a shortened version of the dataset was prepared to contain only the patients’ registration numbers, the order number of the day of hospital stay, and the 14 previously selected features. The sex was coded with numbers 0 (female) and 1 (male). All the feature columns were scaled by calculating the z-score statistics. As a measure of differences, the following distances were considered: Minkowski (including Manhattan and Euclidian distances as special cases), Machalanobis and Spearman [40]. As a result of the search, a list of the most similar patients was formed with indicating the day for which the degree of similarity was maximal.

## Results

### General description of DSS

**Fig. 2:**
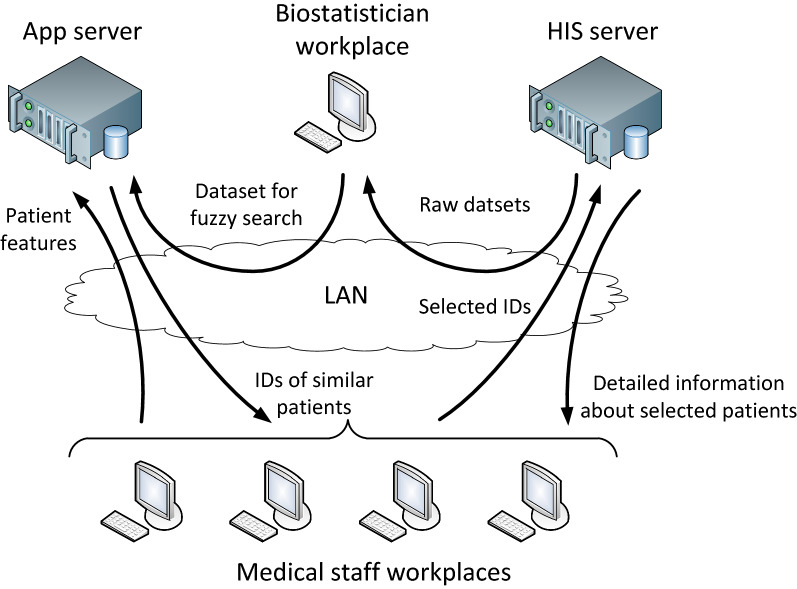
The structure of the developed DSS

The general structural diagram of the developed DSS is shown in Figure 2. An employee of the biostatistics department of the clinic downloads 6 raw datasets from the local HIS, which are further to be combined into a single dataset according to the principle described in Subsection 3.2. Next, the outliers are analyzed manually by finding abnormally large or small values in the data. The detected outliers are discussed with the clinicians for their correctness and the necessity of their exclusion from the dataset. On the cleaned dataset, a list of the most important features is selected, so that doctors are able to search for analogies. Then the shortened version of the dataset is uploaded to the application server. On running the application, it loads the dataset and, based on the available columns, synthesizes the input fields for a graphical user interface (GUI). After entering the features of a patient whose condition the doctor doubts, the GUI displays a brief information on similar cases recorded earlier. Having selected the analogies that deserve the most attention (for example, cases in which a complication has developed), the doctor can obtain a detailed diary of the cases from the HIS using the registration numbers of the patients.

The first test version of the software was based on data from the patients who were hospitalized at the Center for the treatment of the new coronavirus infection at hospital throughout May 2020. Subsequently, the system was updated bi-weekly as the patient data in the local HIS were growing. The current version of the DSS database (as of September 1, 2020) is based on the information on 1572 patients with a median follow-up time of 8 days.

### Selection of main patient features

For the selection of the most important attributes that describe the condition of a patient with COVID-19, we have trained a few machine learning models. The independent variables were the values of the features recorded on a particular day (see Subsection 3.3).

**Fig. 3:**
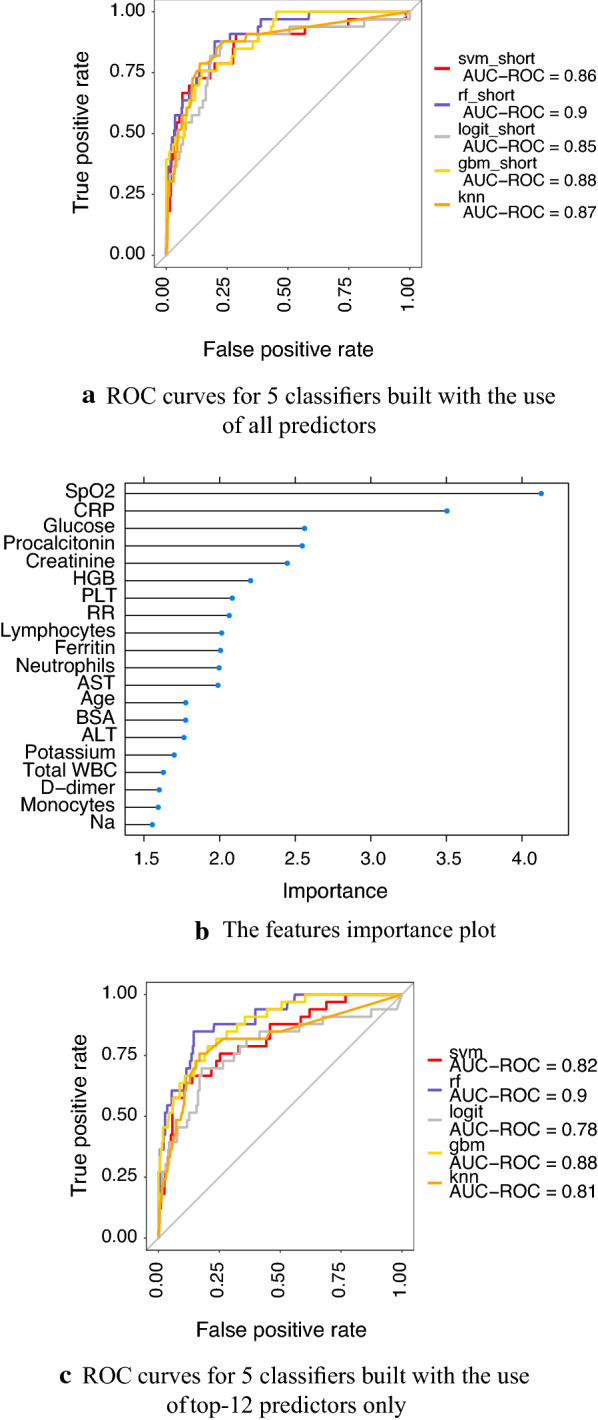
Results of a features selection procedure

The dependent variable was the fact that the patient entered the intensive care unit within a week from the time of taking the tests. The resulting ROC - curves plotted on the basis of all analyzed attributes are shown in Figure 3a. As you can see, the random forest algorithm turned out to be the best in terms of AUC (AUC = 0.9). For this algorithm, the features were ranked according to their importance using the *VarImp* function. The results are shown in Figure 3b (the first 20 attributes are given).

Further on, the minimum number of attributes providing AUC at 0.9 level were selected (their number was 12). The ROC-curves, built using these 12 features, are shown in Figure 3c. As one can see, in the random forest method, the AUC value remained at the level of 0.9, which indicates that the selected features contain a significant amount of information about the patient’s condition. Later, age and sex were added to them as well. Thus, at the moment, the determination of the patient’s current state is made according to the following 14 features:

- demography: age, sex;
- clinical blood test: hemoglobin, as well as the absolute values of platelets, lymphocytes and neutrophils;
- biochemistry: SRP, procalcitonin, ferritin, creatinine, glucose, AST;
- vital indicators: SpO2, RR.

### Graphical user interface

**Fig. 4:**
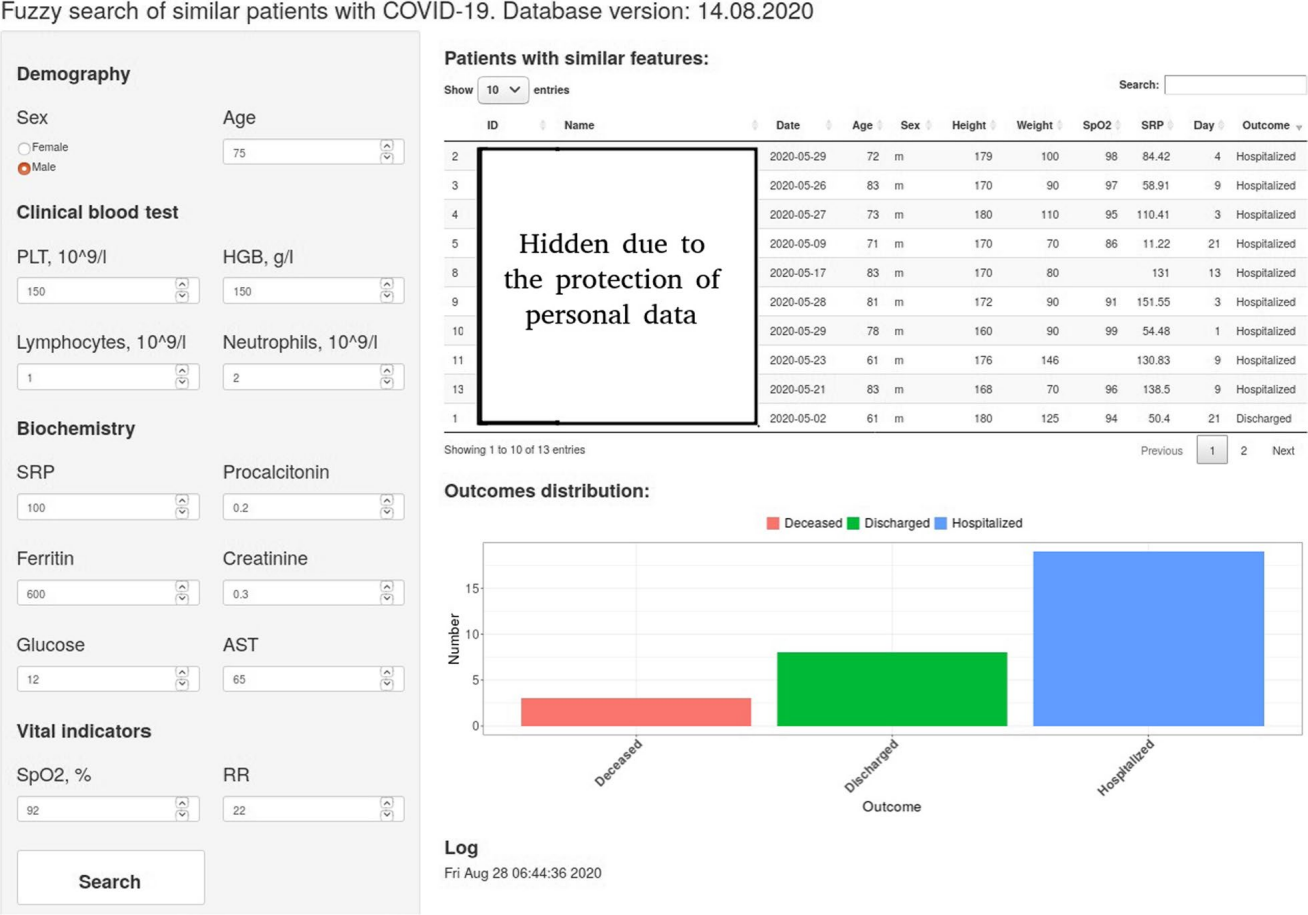
Graphical user interface

Figure 4 shows an example of a graphical user interface. On the left panel there are elements for entering patient data, on the right - the search results in the database. For similar cases found, a brief summary is provided indicating the patient ID, full name, the date on which the entered data match as much as possible, some demographic and clinical laboratory parameters, as well as the outcome of hospital stay. There is also a histogram of outcomes for the detected similar cases, which allows us to quickly navigate for the severity of the patient’s condition. Using the patient’s ID obtained as a result of the search, we can then get a detailed diary of their stay in the hospital from the HIS.

## Discussion

Our experience of deploying the developed system in the hospital showed a great interest in such tools on the part of medical workers. The lack of unambiguous instructions issued by the application helps reduce the level of skepticism and rejection among experienced doctors. At the same time, the need to independently draw conclusions on complex cases based on the study of several case histories improves qualifications and speed of immersion into a new subject area for doctors in related specializations.

Since the application deployment, more than 200 queries were addressed to the DSS. Their analysis showed that our system turned out to be particularly useful in the following three use-cases as stated by physicians.

1. *Hesitation about the application of a medicine with controversial effects.* Particularly we noticed that early application of dexamethasone didn’t prevent patients in moderate conditions from developing severe complications. This finding was later rigorously proved in RECOVERY project [41].
2. *Optimization of a testing plan for a particular patient.* The study of similar cases may help foresee a rapid deterioration of a patient’s condition, which provides good reason for making particular medical tests more frequently.
3. *Forming a pool of patients for a further rehabilitation program.* Observing long-term consequences for similar patients who were discharged earlier, we may add the analyzed patient to the invitation list for a consequent rehabilitation program.

In terms of choice of tools for DSS development in a pandemic, our conclusions are consistent with those of the authors of work [42]: in an unexpectedly changing environment, the choice of tools is determined by their potential for a rapid prototyping using ready-made modules. In this sense, *R* and *Shiny* are one of the best combinations of software. In our case, the development and deployment of the first version of the DSS took no more than a week, which made it possible to begin intensive testing of the proposed approach shortly after the opening of the COVID-19 Treatment Center.

## Conclusion

The study showed a great potential for the application of DSS based on fuzzy search of information in conditions of uncertainty at the time of a pandemic caused by a poorly known pathogen. Our experience points to the possibility of the use of such systems both to support decisions taken by doctors, and to train medical staff. Besides, optimization of a testing plan for the patients may decrease a financial burden on the hospital during a high stress period.

The following future directions for the system improvement may be proposed:

1. Integrate the developed DSS into HIS for the automatization of the entering of features and the extracting of the cases found.
2. Propose advanced vizualization for the data to speed up the comprehensive analysis of the patient state.
3. Extend the search data from instant values to time-series chunks with the potential application of dimension reduction techniques [43].
